# A case report of zinc phosphide poisoning: complicated by acute renal failure and tubulo interstitial nephritis

**DOI:** 10.1186/s40360-017-0144-7

**Published:** 2017-05-25

**Authors:** Nilukshana Yogendranathan, H. M. M. T. B. Herath, Thenuka Sivasundaram, R. Constantine, Aruna Kulatunga

**Affiliations:** National Hospital, Colombo, Sri Lanka

**Keywords:** Zinc phosphide, Run Rat® poisoning, Tubulointerstitial nephritis, Renal failure

## Abstract

**Background:**

Run Rat® is a rodenticide widely used against small mammals. It comprises of a minimum of 32% zinc phosphide which is highly toxic in acute exposures to humans. It may be consumed accidentally or intentionally. It enters the body via skin, respiratory and gastrointestinal tracts. Zinc phosphide is hydrolyzed by the gastric acid and is transformed into phosphine gas. Phosphine is a respiratory toxin that inhibits cytochrome C oxidase system resulting in renal failure and liver failure.

**Case presentation:**

A 35 year old Sri Lankan female presented following ingestion of 2.5 g of Run Rat®, which is a branded preparation of zinc phosphide, resulting in 61 mg/kg poison load. She developed severe acute kidney injury with acute tubular necrosis, subnephrotic ranged proteinuria and tubulointerstitial nephritis for which she underwent haemodialysis three times along with other measures of resuscitation. She also developed elevated liver enzymes with hyperblirubinaemia, hypoalbuminaemia, acute pancreatitis and mild myocarditis. She improved with supportive therapy over a period of 3 weeks.

**Conclusion:**

Run Rat® is a commonly used rodenticide and the toxic effects are mediated through conversion of phosphide to phosphine gas. The majority of the deaths had occurred in the first 12 to 24 h and the main causes identified are refractory hypotension and arrhythmias. The late deaths (beyond 24 h) had been commonly due to adult respiratory distress syndrome, liver and renal failure. The outcome is poorer with delayed presentation, development of coagulopathy, hyperglycaemia and multiorgan failure with elevated liver enzymes. In our patient, Zinc phosphide poisoning caused severe acute kidney injury, abnormal liver profile, pancreatitis and possible myocarditis. The patient improved with repeated haemodialysis. The renal biopsy revealed acute tubulointerstitial nephritis with acute tubular necrosis.

In tropical countries, the rural population engaged in agriculture has easier access to the compound, as it is available at a lower cost. Furthermore, the lack of an antidote and advanced resuscitative measures such as inotropic supportive therapy and renal replacement facilities at most of the peripheral hospitals pose a major challenge in providing timely interventions to prevent deaths.

## Background

Run Rat® is a widely used rodenticide. It is used against small mammals namely mice, rats, field mice, and squirrels^.^. It is commercially available in different compositions and comprises of a minimum of 32% zinc phosphide and the remainder being other ingredients including ammonium carbamate. Zinc phosphide (Zn_3_P_2_) is an inorganic chemical compound that is greyish solid, often dark or even black. The commercial sample consumed in this case report was in a bottle that contained 5 g of Run Rat® as a dark grey crystalline powdered form.

Zinc phosphide is highly toxic in acute exposure to humans. It may be consumed accidentally or intentionally as means of suicidal or homicidal acts. Other routes of entry into the body could be via inhalation or through the skin. Zinc phosphide is hydrolysed by the gastric acid and is transformed into phosphine gas. Phosphine gets absorbed through the stomach and intestine into the blood stream [1]. Ammonium carbamate decomposes into ammonia and carbon dioxide. Phosphine is a respiratory toxin that inhibits cytochrome C oxidase resulting in renal failure and liver failure [2]. Run Rat® poisoning due to self-ingestion is common in Sri Lanka. However severe complications with organ dysfunction had been rarely reported.

Metal phosphide poisoning is fairly common especially in the tropical countries [3]. Both aluminium and zinc phosphide poisoning lead to deleterious outcomes via the phosphine gas mediated effects on cellular respiration [4]. Most of the deaths had been due to refractory hypovolaemia and prerenal kidney injury together with fulminant liver failure, acute pulmonary oedema and metabolic acidosis both high and normal anion gap due to lactic acidosis caused by hypoxia, distal renal tubular acidosis respectively.

This is a case of a 35 year old female who ingested about 2.5 g of Run Rat® poison (61 mg/kg of zinc phosphide) and developed liver, renal and pancreatic toxic manifestations. She developed subnephrotic ranged proteinuria and the renal biopsy showed tubulointerstitial nephritis, along with serum creatinine values of >1000 μmol/L which is not described in the literature previously. She was managed symptomatically and was subjected to haemodialysis three times after which the renal functions began to improve gradually. She was also found to be having markedly elevated liver enzymes reaching beyond 1000 U/L along with high serum amylase levels which also had not commonly been reported in the literature. Almost all the fatal patients had been haemodynamically unfit to undergo haemodialysis. However, this patient was fortunately stable enough to be subjected to haemodialysis during the oliguric phase of the kidney injury.

We decided to report our patient as hardly any case with this degree of severe renal impairment and liver enzyme derangement has been reported in the literature. Furthermore, acute tubulointerstitial nephritis following zinc phosphide had been very rarely reported. The general awareness regarding the management of this toxin among clinicians is lacking in Asian countries. Still no specific antidote has been tried on humans leaving only the previous case reports and case series to aid physicians in treatment [3]. Importance of taking serious precautions against this compound is further emphasized because of the free availability and accessibilities to this cheap rodenticide making this a common poison used in deliberate self harm or attempted suicide.

## Case presentation

A 35 year old female was transferred from a local hospital to our acute medical unit. She is a mother of three children from a coastal area of Sri Lanka. She had ingested about 2.5 g of Run Rat® poison as an impulsive act following a confrontation with her husband. She had been taken to the local hospital immediately by her family where she had been treated for the first 3 days. She has had vomiting, abdominal pain and dizziness on admission. However the haemodynamic parameters at presentation could not be verified since she was haemodynamically stable by the time she was transferred to us.

She was icteric but was otherwise normal on examination. The patient had developed oliguria following admission along with a rise in serum creatinine, which reached over 1000 μmol/L (Table 1). However there was no clinical acidosis. Her liver enzymes and serum bilirubin levels were elevated and the serum albumin was low (Table 1). Coagulation profile was normal (Table 1). Serum amylase was also elevated (Table 1). Complete blood count initially showed a low white cell and platelet counts (Table 1), which later became normal. Blood picture revealed normochromic normocytic cells and a few acanthocytes. She had mild hyponatremia but other electrolytes were within the normal range (Table 1). ESR was 24 mm in the first hour and CRP was <5 mg/L.

**Table 1:** Summary of investigations

Investigation and value	Normal range	Investigation and value	Normal range
WBC 3.57 × 10^3^/μL	4–10	Neutrophils 1.72× 10^3^/μL	2–7
Lymphocytes 0.96 × 10^3^/μL	0.8–4	Platelets 129 × 10^3^/μL	150–450
Haemoglobin 13 g/dL	11–16	Serum sodium 130 mmol/L	135–148
Serum creatinine = 1324 μmil/L	60–120	Serum potassium 4.8 mmol/	3.5–5.1
AST 276 U/L	10–35	ALT 1652 U/L	10–40
Alkaline phosphatase = 476 U/L	100–360	INR 0.96	
APTT 30. 9 (sec)	30–36	Amylase 242 U/L	(22–80)
Albumin 30 g/L	36–50	Globulin 32 g/L	22–40
Total bilirubin μmol/L 109.9	5–21	Direct bilirubin 67.1 μmol/L	<3.4
Ionized calcium 1.21 mmol/L	(1.0–1.3)	Serum magnesium 1.1 mg/dl	(1.7–2.7)
Serum phosphorous 1.0 mmol/L	(0.8–1.5)	Troponin I < 0.1 ng/ml	<0.5
CK-MB < 2 ng/ml	<5		

*WBC* white blood cells, *ALT* alanine transaminase, *AST* aspartate aminotransferase, *INR* international normalized ratio, *APTT* activated partial thromboplastintime

Urinalysis showed pus cells (25–30 per hpf) and RBC (6–8 per hpf). Dysmorphic RBC, cellular casts and eosinophils were not seen in the urine. Urine protein- creatinine ratio was 2.65, which was in subnephrotic range. We were unable to assess 24 h urinary protein excretion. Both C3 and C4 complement levels and antinuclear factor were normal. [Complement C3 = 169 mg/dL (normal range 83–177), Complement C4 = 58 mg/dL (normal range12-86)]. She was negative for HIV 1 & 2 and VDRL. Hepatitis C antibody and Hepatitis B surface antigen were also negative. Thyroid functions were normal.

Ultrasound scan revealed a normal sized liver with uniform echo texture. There was no intra or extra hepatic duct dilatation. Both kidneys were normal in size (Right 11 cm; Left 12.3 cm) but the renal cortical echo texture was increased. ECG showed T inversions in chest leads V1-V4, which was slightly dynamic, but Troponin I, CK MB and myoglobin were within the normal range. 2D echocardiogram did not reveal wall motion abnormalities.

**Fig. 1:**
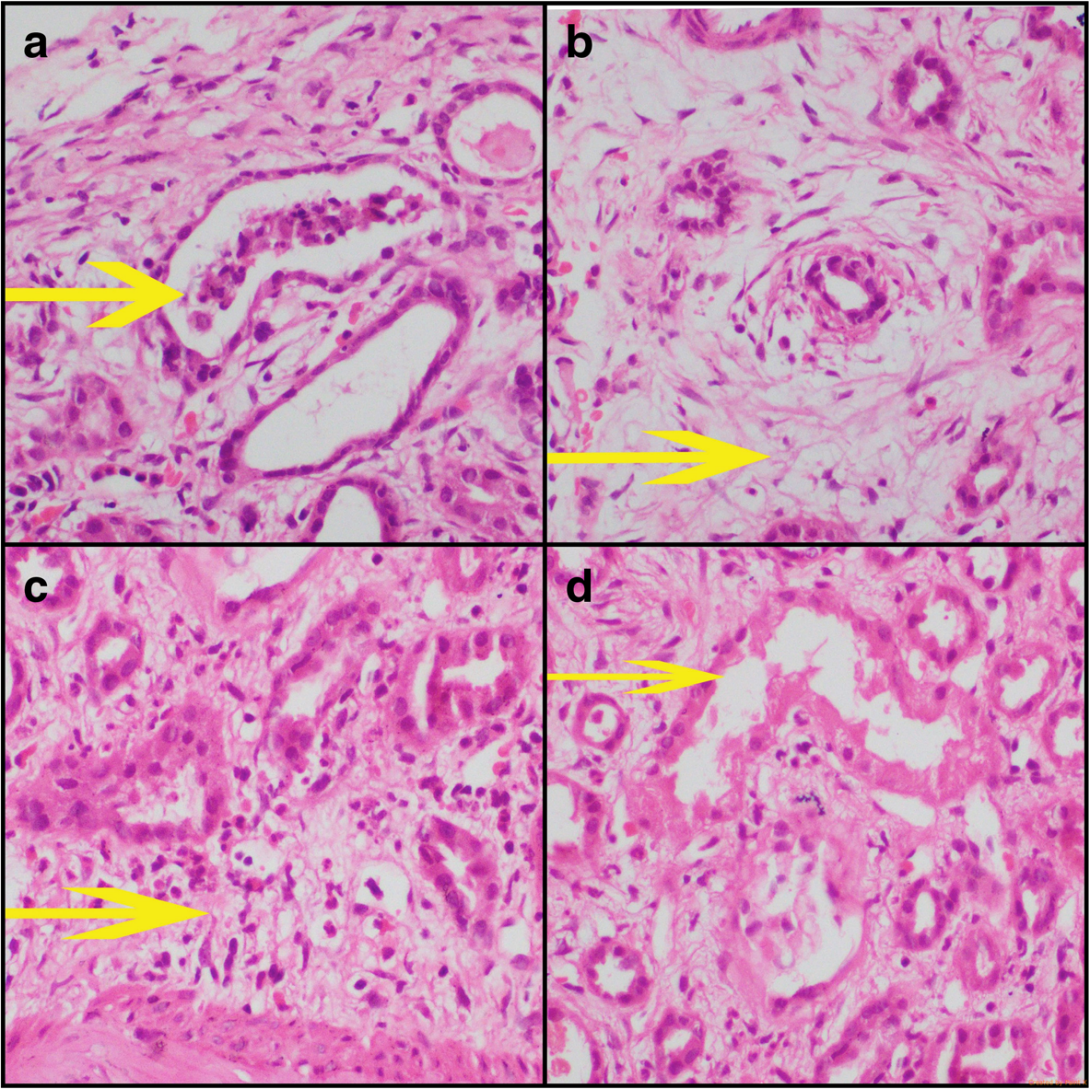
*Yellow arrow* depicts as follows: **a** Damaged renal tubule with flattened out epithelium and containing a granular cast in the lumen. **b** Inflammed oedematous interstitium. **c** Infiltration of interstitium by neutrophils and eosinophils. **d** Necrosed tubules

She was subjected to a renal biopsy which revealed 17 glomeruli that were all viable and histologically unremarkable (Fig. 1). There was no evidence of mesangial hypercellularity, mesangial expansion, glomerular capillary membrane thickening, fibrinoid necrosis, neutrophilic infiltrate, crescent formation or sclerosis. There was mild tubular injury. The cells showed regenerative changes. Some of the tubules contained granular cell casts. The interstitium was oedematous and contained mild to moderate inflammatory infiltrates composed of neutrophils, eosinophils and lymphocytes. Lymphoid aggregates or plasma cells were not seen. The blood vessels were histologically unremarkable.

The patient underwent haemodialysis three times and there after the renal impairment began to improve. The serum creatinine gradually reduced over 3 weeks to around 90 μmol/L and the liver enzymes, serum bilirubin along with serum amylase levels improved with the supportive therapy gradually.

## Discussion

Zinc phosphide poisoning is recognised as a significant cause of morbidity and mortality among both socioeconomically low but economically active age demographics, especially in developing countries. A dosage of 4 to 5 g of zinc phosphide (55–70 mg/kg) had resulted in human deaths in acute toxicity [1]. Our patient developed acute tubulointerstitial nephritis with subnephrotic range proteinuria, elevated hepatic enzymes with hyperbilirubinaemia, acute pancreatitis and dynamic ECG changes possibly due to an element of myocarditis following ingestion of 2.5 g (61 mg/kg) of zinc phosphide.

The common symptoms of zinc phosphide poisoning following inhalation are abdominal pain, cough, dizziness, headache, sorethroat and laboured breathing. The symptoms following ingestion are nausea, vomiting, abdominal pain, chest tightness, agitation, cyanosis, loss of consciousness and convulsions.

Lauterbach M et al., have concluded at the end of a study done in Germany analyzing a total number of 188 cases presenting with hydrogen phosphide poisoning, that most of the hydrogen phosphide poisoning was unintentional. They further commented that the severity of symptoms differs between intentional and unintentional phosphide poisoning and that careful monitoring is needed to those presenting with intentional phosphide poisoning [5]. Congestive cardiac failure, adult respiratory distress syndrome, oliguric acute kidney injury (in about 50% patients), fulminant hepatic failure with elevated liver enzymes warranting liver transplant, thrombocytopaenia, metabolic acidosis, distal rental tubular acidosis, hypomagnesaemia, severe hypocalcaemia, hypokalaemia, intravascular haemolysis, disseminated intravascular coagulation and generalized tonic clonic seizures with delirium have all been recognized with phosphide poisoning [4]. It also causes myocarditis and pericarditis with ECG changes such as sinus tachycardia, bradycardia, supraventricular ectopics, ventricular ectopics, atrial fibrillation, ventricular fibrillation, conduction defects namely wide QRS complex, A-V conduction defects, bundle branch block, complete heart block and ST-T changes such as ST depression, ST elevation, T wave changes [3, 4].

The mortality rate of zinc phosphide poisoning is around 37–100% [3]. It causes both metabolic and non metabolic toxic effects. The mechanism of phosphide poisoning has been explained as follows by various studies.

1. Phosphine inhibits the oxygen uptake in the rat liver mitochondria [6].
2. It inhibits ADP uncoupled site and ion stimulated respiration thus affecting pyruvate malate, succinate, glycerophosphate and ascorbate cytochrome biomolecules in liver mitochondria [7]. However the exact target site is a contentious issue.
3. It alters mitochondrial morphology, inhibits oxidative respiration by 70% and causes a large drop in mitochondrial membrane potential within 5 h of exposure [8].
4. Phosphine and hydrogen peroxide can interact to form the highly reactive hydroxyl radical that causes lipid peroxidation which is the main mechanism of oxidative damage to cell structures that lead to cell death [3].
5. Cytochrome C oxidase system is inhibited [2, 9].
6. There is decreased activity of cytochrome oxidase along with altered NADH and succinic dehydrogenase activities [10].
7. It increases the lipid peroxidation in the central nervous system while reducing the antioxidant defence system such as superoxide dismutase, catalase and glutathione reductase [11].
8. It inhibits protein synthesis and enzymatic activity
9. It has anti-choline esterase effects and also causes denaturation of oxy-haemoglobin molecules [12].

Phosphide poisoning is also known to cause glycaemic derangement. Severe hypoglycaemia is more commonly seen due to the reduced hepatic glycogenolysis and gluconeogenesis. However, interestingly a transient hyperglycaemia that could rarely occur is possibly due to pancreatic involvement. It is concluded in the literature that treatment of hyperglycaemia reduces the cellular oxygen consumption with re-entry of glucose into the intracellular compartment. Gunaratne et al., had suggested that the delayed presentation and having a hyperglycaemia that could rarely occur due to unexplained mechanisms, predict a poorer outcome [13]. Jain et al., have also concluded that one should look for hyperglycaemia which warrants timely correction [14]. Mehrpour et al., have added reinforcement to this fact at the end of an analytical study of aluminium phosphide poisoning in that the patients who had died had relatively significantly higher glucose values than those who had survived [15].

Surgit Singh et al., have concluded that the outcome of phosphide poisoning can be correlated with the number of induced vomiting the patient gets, severity of hypovolaemia and acidosis [9]. Majority of the deaths had occurred in the first 12 to 24 h and the main causes identified are refractory hypotension and arrhythmias. The late deaths (beyond 24 h) had been commonly due to adult respiratory distress syndrome, liver failure and renal failure. Furthermore the outcome is poorer with delayed presentation, development of coagulopathy, hyperglycaemia and multiorgan failure [15].

Dogan et al., had reported a 21-year-old female who had presented with toxic level of zinc phosphide poisoning and deteriorated into severe hypovolaemic shock and acidosis making her unfit for haemodialysis and expired while awaiting bedside haemofiltration. Mostly the victims succumbed despite of fluid resuscitation, inotropic support, renal replacement or bicarbonate therapy [3]. Ozgur et al., had reported another 22 year old patient who expired following severe refractory acute pulmonary oedema with renal as well as liver impairment, myopericarditis leading to refractory acidosis and respiratory arrest. The autopsy had revealed congested liver, lungs and spleen [16]. Churgh and Aggarwal., had reported twenty cases of zinc phosphide poisoning who had presented between years 1992 to 1996 in India. All the victims had vomiting and abdominal pain; the majority had autonomic symptoms (80%), dyspnoea (75%) and metabolic acidosis (60%). Forty Percent of patients had suffered hypotension with shock out of which 25% patients had died [17].

Gunaratne et al., have recently reported a case of zinc phosphide poisoning in the Ceylon Medical Journal. This patient also has had liver and renal derangement similar to our patient. However the degree of severity had been to a relatively lesser extent. The liver enzymes had risen to ALT 157 U/L and AST 93U/L; serum creatinine 145 μmol/L. This patient had deteriorated into haemorrhagic acute pulmonary oedema and developed asystolic cardiac arrest while being ventilated. She had been haemodynamically unstable to undergo haemodialysis unlike our patient [13].

Saleki et al., had analysed the histologic changes that manifest in the liver of 38 cases of fatal phosphine poisonings. The following were listed out as the main histologic alterations: sinusoidal congestion; 12 cases (31.6%), severe sinusoidal congestion; 25 cases (45.8%), central vein congestion; 23 cases (60.5%), centrilobular necrosis; 3 cases (7.9%), hepatocytes nuclear fragmentation; 6 cases (15.8%), sinusoidal clusters of polymorphonuclear leukocytes; 12 cases (31.6%), and mild macrovesicular steatosis; 5 cases (13.2%) [18]. Resistant hypotension and resistant metabolic acidosis have been the two main well recognized causes of death in most of the cases reported [3]. However Gokdemir et al., have concluded that the mortality rate is twice as higher in the patients who develop elevated hepatic enzymes following zinc phosphide poisoning [19]. The metabolic acidosis could occur due to hypoxia induced lactic acid production, renal and liver impairment leading to fatal outcomes.

There have been very rare instances where renal biopsy had been performed in the survivors of zinc phosphide poisoning. However there are few cases reports including autopsy findings of the renal tissue. Most of the patients had developed acute tubular necrosis especially proximal convoluted tubules in a patchy distribution probably due to prerenal hypoxic insult [20]. Our patient had mainly tubular inflammation, granular casts associated with interstitial infiltration by neutrophils, eosinophils and lymphocytes. There was evidence of acute tubular necrosis as well, mainly in the regenerative phase as the biopsy was performed at the end of 2 weeks from the initial presentation.

On analysis of the predictive factors of prognosis as discussed above our patient did not have a delay in presenting to seek treatment, was hydrated adequately and did not have hyperglycaemia despite of the pancreatic involvement. Furthermore, the renal impairment was amenable to renal replacement therapy as she was haemodynamically stable unlike in most of the cases reported in the literature.

Management is merely symptomatic and supportive as there is no definite antidote against phosphides. Adequate fluid resuscitation via oral and intravenous routes is essential. Inotropic support with Dobutamine and further hydrocortisone or dexamethasone is recommended if the response is poor despite fluid resusucitation. Vitamin K (phytomenadione) can be given if the Prothrombin time is prolonged [21]. A definite selection criterion for liver transplantation for patients who deteriorate into acute fulminant hepatic failure is yet to be studied. However a study held in Kerala has suggested that the presence of a MELD score of 31 on the sixth day or the presence of encephalopathy at any time after ingestion is a strong predictor of mortality without a liver transplant [6].

The problems encountered in Asian countries are that such rodenticides are abundantly available although its usage is illegal. The tropical countries and the rural population engaged in agriculture have easier access to the compound, as it is available at a lower cost. Furthermore, the lack of an antidote and advanced resuscitative measures such as inotropic supportive therapy and renal replacement facilities in most of the peripheral hospitals make the management of victims presenting with a lethal dose a major challenge.

## Conclusion

The clinical manifestations of zinc phosphide poisoning are nausea, vomiting, abdominal pain, chest tightness, agitation, cyanosis, loss of consciousness and convulsions. The major complications are congestive cardiac failure, adult respiratory distress syndrome, oliguric acute kidney injury, fulminant hepatic failure with elevated liver enzymes warranting liver transplant, thrombocytopaenia, metabolic acidosis, distal renal tubular acidosis, hypomagnesaemia, severe hypocalcaemia, hypokalaemia, intravascular haemolysis, disseminated intravascular coagulation, generalized tonic clonic seizures and delirium

Majority of the deaths had occurred in the first 12 to 24 h and the main causes identified are refractory hypotension and arrhythmias. The late deaths (beyond 24 h) had been commonly due to adult respiratory distress syndrome, liver failure and renal failure. The outcome is poorer with delayed presentation, development of coagulopathy, hyperglycaemia and multiorgan failure with elevated liver enzymes.

In Asian countries zinc phosphide is abundantly available at a lower cost. Thus it is a commonly used compound in suicides. Management is merely symptomatic and supportive as there is no definite antidote against phosphides. Thus the lack of specific antidote and inadequate resuscitative therapy like renal replacement at peripheral hospitals and delay in transport to tertiary medical centres pose a great challenge to the physicians. Measures should be taken to educate the rural population on the fatal complications of such poisoning and update the knowledge of medical personnels at peripheral hospitals on timely management of life threatening end organ damage. Furthermore the resuscitation therapy at local hospitals and mode of transferring system to tertiary centres warrant improvement to prevent deaths due to major organ damage.

## Abbreviations

ALT: Alanine transaminase
AST: Aspartate aminotransferase
ECG: Electrocardiogram
ESR: Erythrocyte sedimentation rate
NADH: Nicotinamide adenine dinucleotide (reduced form).
